# Sealing Ability of AH Plus and GuttaFlow Bioseal

**DOI:** 10.1155/2020/8892561

**Published:** 2020-09-22

**Authors:** Soo-Hyuk Lee, Soram Oh, Adel Saeed Al-Ghamdi, Ayman Omar Mandorah, Kee-Yeon Kum, Seok Woo Chang

**Affiliations:** ^1^Department of Conservative Dentistry, Kyung Hee University, School of Dentistry, 23 Kyungheedaero, Dongdaemun-gu, Seoul 02447, Republic of Korea; ^2^Department of Conservative Dentistry, Kyung Hee University, Dental Hospital, 23 Kyungheedaero, Dongdaemun-gu, Seoul 02447, Republic of Korea; ^3^Department of Dentistry, King Abdulaziz Hospital, MOH, Jeddah 22421, Saudi Arabia; ^4^Department of Endodontics, Restorative and Dental Materials, Faculty of Dentistry, Taif University, Taif 26571, Saudi Arabia; ^5^Department of Conservative Dentistry, Dental Research Institute, National Dental Care Center for Persons with Special Needs, Seoul National University Dental Hospital, Seoul National University School of Dentistry, Seoul 03080, Republic of Korea

## Abstract

The objective of root canal obturation is to achieve a fluid-tight seal. Recently, GuttaFlow bioseal (GB), a root canal sealer composed of polydimethylsiloxane, gutta-percha particles, and bioactive glass ceramics, has been developed, to enhance the sealing ability of root canal filling material. The objective of this study was to assess the sealing ability of GB using a subnanoliter-scaled fluid-flow measuring device and to compare with that of AH Plus (AH). The fluid flow in root canal-filled teeth using either gutta-percha cone (GP) with AH (GAR; *n* = 10) or GP with GB (GBR; *n* = 10) and in GP inserted in AH blocks (GA; *n* = 10) or GP inserted in GB blocks (GB; *n* = 10) was measured. In addition, fluid flow in sealer blocks of AH (A; *n* = 10) and GB (B; *n* = 10), which served as negative controls, was measured. Root canal-filled teeth using GP without any sealer (GR) acted as positive controls (*n* = 10). The leakage was obtained by calculating the volume of moved water by time (s), after stabilization of the fluid flow was achieved. Statistical analysis was performed using the Kruskal–Wallis test and Mann–Whitney *U*-test with Bonferroni correction. A *p* value less than 0.00238 (0.05/21) was considered significantly different. The mean leakages (nL/s) in the groups are as follows: GAR, 0.0958 ± 0.0543; GBR, 0.0223 ± 0.0246; GA, 0.0644 ± 0.0803; GB, 0.0267 ± 0.0182; A, 0.0055 ± 0.0057; B, 0.0052 ± 0.005; and GR, 0.2892 ± 0.3018. The mean leakage in the GBR group was lower than that in the GAR group (*p* = 0.001), while the mean leakages in the GA and GB groups were not significantly different. GuttaFlow bioseal can be useful in single-cone obturation technique.

## 1. Introduction

Root canal treatment is the process of cleaning, shaping, and obturating the root canal system. Minimizing leakage and achieving fluid-tight seal are as important as complete cleaning and shaping for the success of root canal treatment [1]. Generally, gutta-percha cone (GP) is used with a root canal sealer for obturating the root canal. Leakage from root-canal fillings can initiate from the materials themselves or from the interfaces between the materials and tooth [2].

Traditional GP with sealer did not provide an impervious seal of the root canal system; therefore, new obturation materials and techniques have been developed over the past decades to obtain an optimum seal in the root canal system [3, 4]. GuttaFlow bioseal (Coltène/Whaledent, Altstätten/Switzerland) is a recently developed, silicone-based, cold-filling sealer containing GP powder and bioactive glass. The manufacturer has claimed that GP combined with bioactive glass can form hydroxyapatite crystals of the surface [5].

Various in vitro experimental methods are used to assess leakage in root canal-filled teeth. These methods include dye penetration, spectrometry of radioisotopes, bacterial penetration, and sectioning followed by microscopic examination [2, 6–9]. The shortcomings of these methods are the lack of reproducibility and semiquantitative nature of the obtained data [9, 10]. Traditional fluid-transport methods measured leakage quantitatively, yet previous methods measured the amount of fluid up to the micrometer or millimeter unit [2, 11, 12]. Those methods lacked accuracy, and leakage measurements had to be performed for a long term to obtain data. Previous studies have analyzed dentinal fluid flow in real time using a subnanoliter-scaled fluid-flow measuring device (NFMD) [13, 14]. This device can measure leakage at subnanoscale and can provide a reproducible method for measurements without destroying the tooth. The aim of this study was to measure the leakage at the interface of GuttaFlow bioseal and GP and that of GuttaFlow bioseal and root dentin using the NFMD and to compare the sealing ability of GuttaFlow bioseal with the AH Plus root canal sealer.

## 2. Materials and Methods

The teeth used in this study were obtained in accordance with the protocol approved by the Institutional Review Board (KH-DT19028, Kyung Hee University Dental Hospital, Seoul, Republic of Korea). The materials used in this study were the AH Plus (Dentsply DE Trey, Konstanz, Germany) and GuttaFlow bioseal root canal sealers and GP points (Meta Biomed, Cheongju, Republic of Korea). The compositions of the materials are described in Table 1.

**Figure 1:**
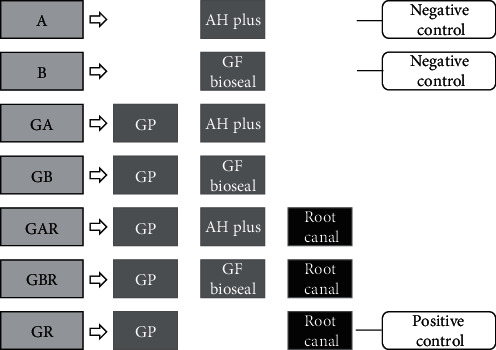
Experimental setup and groups. Experimental groups consisted of root canal-filled teeth with GP cone and sealer and a GP cone-inserted sealer mass. Positive control groups consisted of root canal-filled teeth with GP cone, and sealer masses acted as negative controls.

The leakages were measured in root canal-treated teeth filled using either GP with AH Plus (GAR; *n* = 10) or GP with GuttaFlow bioseal (GBR; *n* = 10) and GP inserted within AH Plus (GA; *n* = 10) or GP inserted within GuttaFlow bioseal (GB; *n* = 10). In addition, leakages were measured in the sealer blocks made of AH Plus (A; *n* = 10) and GuttaFlow bioseal (B; *n* = 10), which served as negative controls. Root canal-filled teeth with GP without any sealer acted as positive controls (GR; *n* = 10). All experimental groups are described in Figure 1.

### 2.1. Leakage Measurements Using NFMD

#### 2.1.1. Specimen Preparation

Extracted human mandibular premolars with single root canals were collected to evaluate the sealing ability of the single-cone technique using the AH plus and GuttaFlow bioseal. The roots were sectioned to a standard length of 8 mm. The #10 K-file (Dentsply Maillefer, Ballaigues, Switzerland) was inserted into the root canal until the file tip was just visible at the foramen, and the working length was calculated by subtracting 0.5 mm from this distance. The root canal was prepared using ProTaper Gold nickel-titanium files (Dentsply Maillefer) sequentially up to F4, while irrigating the canals using 5.25% sodium hypochlorite. After completion of canal shaping, the root canal was irrigated with 17% EDTA for 1 minute to remove the smear layer. Thereafter, the root canal was flushed with saline and was dried using paper points. Thirty roots were allocated to the following 3 groups: 2 experimental groups and 1 positive control group. For root canals in the GAR group, AH Plus was mixed according to the manufacturer's instructions and a GP point of size #40/06 was coated with AH Plus and was inserted in the root canal to the complete working length. The excess GP was cut using a heat carrier (SuperEndo Alpha ΙΙ; B & L Biotech, Ansan, Republic of Korea) at the level of the root canal orifice according to the usual protocol of the single-cone technique [15, 16]. For root canals in the GBR group, the mixing tip of GuttaFlow bioseal was placed within the root canal, and approximately half of the canal was filled with GuttaFlow bioseal. A #40/06 size GP point was lightly coated with the sealer and was slowly inserted into the canal up to the working length. The excess GP was cut using SuperEndo Alpha ΙΙ at the level of the root canal orifice [17]. For root canals in the positive control group (GR), the #40/06 GP point was inserted into the canal without any sealer and was cut with a heat carrier at the level of the root canal orifice.

**Figure 2:**
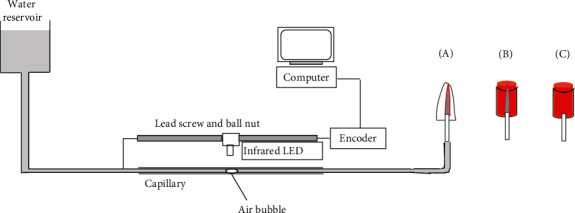
A schematic diagram of a subnanoliter-scaled fluid-flow measuring device connected to a root canal-treated tooth (A), a GP cone-inserted sealer mass (B), and a block of sealer (C).

After being allowed to set in a chamber with 100% humidity at 37°C for 7 days, 1 mm of GP at the orifice was removed, and a sandblasted metal tube was inserted into the cervical orifice of the root canal to 2 mm depth (Figure 2(a)). The space between the metal tube and root dentin was filled with a flowable composite resin (G-aenial Flo, GC, Tokyo, Japan) following application of a dentin-bonding agent (All Bond Universal, Bisco Inc, Schaumburg, IL, USA). All external surfaces of the root specimen with the metal tube and composite plug except 2 mm of the tip were covered with a nail varnish.

To evaluate leakage between the GP and sealer, specimens in the GA and GB groups were combinations of GP and sealer without root dentin. The root canal sealer (AH Plus or GuttaFlow bioseal) was mixed according to the manufacturer's instructions. Thereafter, the sealer was injected into a cylinder-shaped plastic mold with 5 mm diameter and 8 mm length, and a #40/06 GP cone was inserted in the center of the sealer paste immediately. After storing in a chamber with 100% humidity at 37°C for 7 days, 1 mm of the coronal end of the GP cone was removed followed by insertion of a metal tube (Figure 2(b)). The space between the tube and sealer was filled with an epoxy adhesive (Uhu GmbH & co., Bühl, Germany). Apical 1 mm of the plastic mold was removed using a surgical blade. All surfaces of the specimen were covered with a nail varnish except the apical 1 mm area.

For specimens in the negative control groups (A and B), either AH Plus or GuttaFlow bioseal was mixed and filled in the plastic mold. After storing in a chamber with 100% humidity at 37°C for 7 days, manipulation of the specimen and coating with a nail varnish were performed in the same manner as for groups GA and GB (Figure 2(c)).

#### 2.1.2. Leakage Measurements Using the NFMD

The metal tube inserted in each specimen was connected to an NFMD (Nano-flow; IB system, Seoul, Republic of Korea), which measures the flow of fluids. A distilled water- (DW-) filled glass capillary (internal diameter: 0.5 mm) was connected between a water reservoir and the specimen (Figure 2). The flow rate was measured for 600 s at a pressure of 50 cm of H_2_O at 21°C. An air bubble was introduced into the capillary, by which the flow of DW could be detected by using a photosensor. The movement of the bubble and the volume of moved DW were measured by computer software. The leakage was obtained by the flow rate, which was calculated by dividing the volume of moved DW by time (s). The flow rate was measured for 5 minutes after stabilization was achieved (nL/s).

#### 2.1.3. Statistical Analysis

Statistical analysis was performed using SPSS software (ver. 19.0.0; IBM Corp., Armonk, NY, USA). The data (flow rate) were not normally distributed and were not satisfied with homogeneity of variance, and the nonparametric test was used. The Kruskal–Wallis test was performed to assess whether flow rates were different among the groups. The Mann–Whitney *U*-test with Bonferroni correction was used for pairwise comparisons. Twenty-one tests were conducted for intergroup pairwise comparisons, and a *p* value less than 0.00238 (0.05/21) was considered significantly different.

### 2.2. Specimen Preparation for the Scanning Electron Microscopy (SEM) Study

After the leakage test, two specimens in groups GAR and GBR were subjected to SEM examination. Using a high-speed saw (IsoMet 5000; Buehler, Lake Bluff, IL, USA), the root was sectioned perpendicular to its long axis to obtain a section of 1.5 mm thickness. The specimens were dried according to the protocol suggested by Perdigao et al. [18]. The specimens were etched with 37% phosphoric acid for 15 seconds and rinsed, followed by fixation with 2.5% glutaraldehyde solution for 12 hours. Thereafter, the specimens were rinsed with 20% phosphate-buffered saline. Furthermore, they were dehydrated in ascending grades of ethanol (25% for 20 minutes, 50% for 20 minutes, 75% for 20 minutes, 95% for 30 minutes, and 100% for 60 minutes). Subsequently, the specimens were immersed in hexamethyldisilazane for 10 minutes. All specimens were platinum-coated before observation under the SEM (Hitachi S-4700; Hitachi, Tokyo, Japan). The interfaces between sealer, dentin, and GP were observed.

## 3. Results

### 3.1. Leakage Measurements Using the NFMD

**Figure 3:**
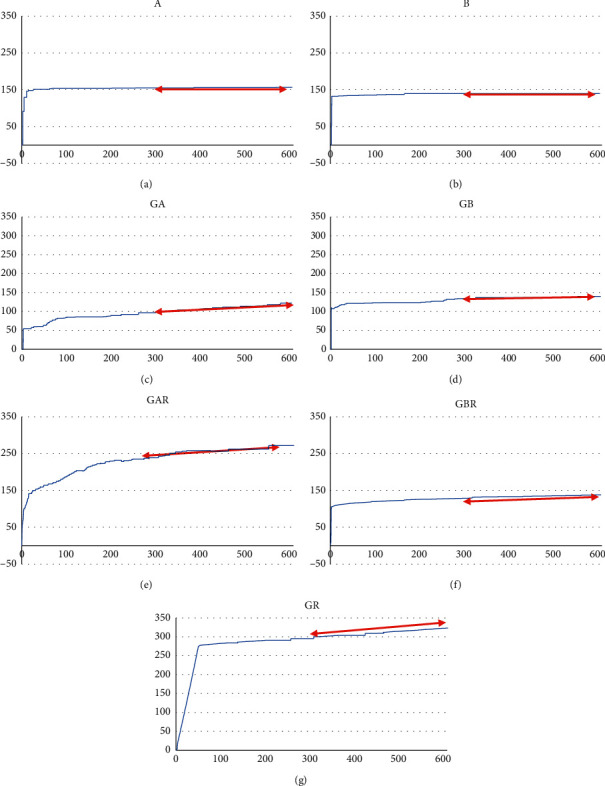
Representative graph of fluid flow (nL) in 600 seconds. Leakage was measured as the flow rate (slope of the graph) after flow stabilization (red arrows). A, AH Plus; B, GuttaFlow bioseal; GA, GP cone with AH Plus; GB, GP cone with GuttaFlow bioseal; GAR, root canal filling with GP cone and AH Plus; GBR, root canal filling with GP cone and GuttaFlow bioseal; and GR, root canal filling with GP cone without sealer.

**Figure 4:**
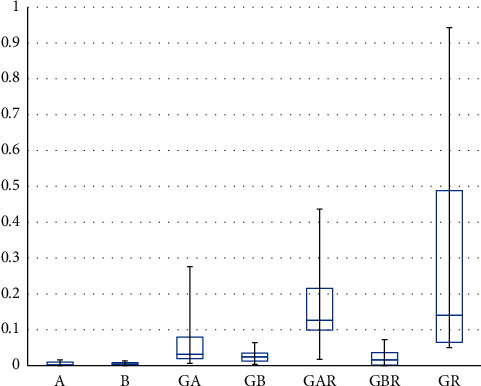
Box and Whisker plot of the leakages (nL/s).

A graph representative of fluid flow in each group is shown in Figure 3, and the median and interquartile range of leakages are shown in Table 2 and Figure 4. The leakage was determined as the slope of fluid flow (nL) to time (s) graph, after the stabilization of fluid flow. The negative control groups (groups A and B) showed the lowest leakage, and no statistically significant difference was observed between the two sealers (*p* = 0.912). No significant differences in leakage were observed among the GBR, GB, and GA groups.

Root canal fillings performed using GP and GuttaFlow bioseal yielded less leakages compared to those performed using GP and AH Plus (*p* = 0.001). No significant differences were observed in leakage among the GBR and negative control groups, i.e., groups A and B (*p* = 0.247 and *p* = 0.19, respectively). Root canal filling performed using GP alone without sealer (positive control) showed the highest leakage (Figure 4).

### 3.2. SEM Examination

**Figure 5:**
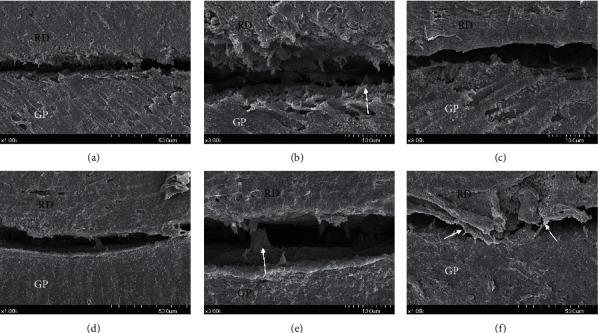
Representative scanning electron microscopy images of root canal-filled specimens. (a) Root canal filling with GP and AH Plus, (b) magnified view of (a) sealer is evident on the root dentin (asterisk) and GP (arrow), and (c) root canal filling with GP and AH Plus, and the root dentin is not coated with sealer. (d) Root canal filling with GP and GuttaFlow bioseal, (e) magnified view of (d) sealer is evident on the root dentin (asterisk) and GP (arrow), (f) root canal filling with GP and GuttaFlow bioseal, and GuttaFlow bioseal connecting the root dentin area and GP (arrows). RD, root dentin; GP, gutta-percha point.

In the SEM images, GP and root dentin in root canals where the sealer was applied on the surface were observed as separate phases (Figures 5(a), 5(b), 5(d), and 5(e)). Some GAR specimens demonstrated areas of root dentin that were not coated by AH Plus (Figure 5(c)). In some specimens, the GuttaFlow bioseal appeared intertwined from the areas covering the dentinal wall to the areas coating the GP (Figure 5(f)).

## 4. Discussion

A number of methods and materials have been developed to achieve a fluid-tight seal of the root canal system for optimum results after endodontic treatment; however, none has been successful in achieving a complete seal [11, 12, 19, 20]. In this study, we observed that GuttaFlow bioseal, a new silicone-based sealer, showed less leakage than AH Plus, when used for root canal filling with the single-cone obturation technique. The lesser leakage of GuttaFlow bioseal compared to AH Plus could be attributed to the volumetric changes that occur during the setting of sealers. According to Tanomaru-Filho et al., GuttaFlow bioseal undergoes 0.14% expansion after storage in distilled water for 7 days, and 0.68% volume contraction after 30 days of storage [21]. They also evaluated the dimensional changes in AH Plus after 7 and 30 days, which were 0.5% expansion and 0.19% contraction, respectively. Camargo et al. reported that the dimensional change in GuttaFlow bioseal after storage in distilled water for 30 days was 2.1% expansion and that in AH Plus was 0.06% expansion [22]. In this study, the flow rate was measured after 7 days of storage in 100% relative humidity. We speculate that volumetric expansion could have increased due to water sorption by GuttaFlow bioseal. The high water sorption ability of GuttaFlow bioseal has been reported previously [23].

No study has measured leakage from the dentin-sealer interface and sealer-GP interface separately. Lee et al. compared the tensile bond strength of sealers against dentin and GP separately [24]. In their study, sealers showed varying degrees of adhesion to GP or dentin, depending on their type. The AH-26 sealer used in their study, an epoxy resin-based sealer similar to AH Plus, showed stronger adhesion to GP than dentin. In our study, the mean leakage in the GAR group was greater than that in the GA group, although the difference was not statistically significant. As the dentin-sealer interface was present in the GAR group, and not in the GA group, the dentin-sealer interface showed leakage. This result could be attributed to the hydrophobicity and shrinkage of AH Plus. The GP-AH Plus sealer interface is extremely hydrophobic [23, 25], while as dentin is a hydrophilic substrate, the dentin-sealer interface would be relatively hydrophilic, acting as a major pathway of leakage. Some SEM images also showed that the AH Plus sealer did not coat the dentinal wall (Figure 5(c)).

SEM examination was performed to examine the sealer-GP and sealer-dentin interfaces. The specimens were dehydrated according to the protocol suggested by Perdigao et al. However, gaps between the materials were evident during specimen preparation. Gaps between the sealer and dentin should be interpreted carefully [26]. As measurements of gap widths would not have obtained statistically significant data, we focused on observing the shape and characteristics of the gaps. The images of SEM examination were randomly selected to represent the interface of each specimen. In case of GuttaFlow bioseal, some precipitate was observed on the tag (Figures 5(d)–5(f)). This precipitate was determined to be bioactive glass, which could be a component of the inherent composition of GuttaFlow bioseal, or a mineralization product formed during the setting process [27]. Further elemental studies should be performed to identify the precipitate, and long-term studies are required to determine the mineralization capacity of bioactive glass.

In the present study, root canal filling performed using GuttaFlow bioseal did not show fluid-tight seals, and the flow of DW was evident (Table 2, Figure 4). No previous study has assessed the sealing ability of GuttaFlow bioseal using fluid flow. According to De-Deus et al., who assessed the leakage of other silicone-based sealers, GuttaFlow (Coltène/Whaledent AG), a silicone-based sealer, showed less leakage than AH Plus or Pulp Canal Sealer EWT [28]. Akcay et al. evaluated dentinal tubule penetration of root canal sealers by using confocal microscopy and did not observe any significant difference in dentinal tubule penetration between GuttaFlow bioseal and AH Plus [16]. Further long-term studies to measure the volumetric changes in GuttaFlow bioseal and water sorption of GuttaFlow bioseal are required to correlate those characteristics and the sealing ability of GuttaFlow bioseal.

The limitation of the present study is the preparing method of SEM specimens. There is a risk that sectioning of the filled root may result in tearing or smearing of GP and sealer, and the vacuum desiccation process could possibly cause an interfacial gap between different materials. An environmental SEM (ESEM) study will be useful to examine the hydrated root canal because ESEM permits the imaging of a wet sample without prior specimen preparation.

## 5. Conclusions

Within the limitation of this study, GuttaFlow bioseal provided more fluid-tight seal than AH Plus when used with the single-cone obturation technique. The interface between GP and one of the tested root canal sealers, i.e., GuttaFlow bioseal and AH Plus, possesses some leakage, which was greater than the negative controls. Further clinical studies need to be conducted regarding the treatment outcomes of the single cone obturation technique with GuttaFlow bioseal.

## Figures and Tables

**Table 1:** Materials used in this study.

Material	Composition	Manufacturer
AH Plus	Paste A: Diglycidil-bisphenol-A-ether, calcium tungsten, zirconium oxide, aerosol, and iron oxide Paste B: Amina-1-adamantane, N, N-dibenzyl-5-oxanonandiamine-1, 9, TCD-di-amine, calcium tungsten, zirconium oxide, and silicone oxide	Dentsply DE Trey, Konstanz, Germany

GuttaFlow bioseal	Gutta-percha powder, polydimethylsiloxane, platinum catalyst, zirconium dioxide, silver (preservative), coloring, and bioactive glass ceramic	Coltène/Whaledent AG, Altstätten/Switzerland

Gutta-percha points	Gutta-percha, zinc oxide, barium sulfate, and calcium carbonate	Meta biomed, Cheongju, Republic of Korea

**Table 2:** Median and interquartile range (IQR) of leakages.

Group	Composition	Leakage (nL/s)	
		Median	IQR
A	AH Plus	0.00265^a^	0.00811
B	GuttaFlow bioseal	0.00474^a^	0.00808
GA	GP cone with AH Plus	0.03146^b,c^	0.06000
GB	GP cone with GuttaFlow bioseal	0.02470^b^	0.02298
GAR	Root canal filling with GP cone and AH Plus	0.12608^c^	0.11628
GBR	Root canal filling with GP cone and GuttaFlow bioseal	0.01615^a,b^	0.03504
GR	Root canal filling with GP cone without sealer	0.14045^c^	0.42347

Different superscript letters indicate a statistically significant difference (*p* < 0.00238).

## Data Availability

The data used to support the findings of this study are available from the corresponding author upon request.
